# Optimizing neuromuscular symmetry through load-progressive closed-chain upper limb training: An EMG-based approach

**DOI:** 10.1016/j.jsampl.2025.100133

**Published:** 2025-12-24

**Authors:** Rafid Qaduri, Taha Jasim, Hiba Hamadi, Safaa Ismaeel, Shaimaa Shihab, Mohammed Al-Jahni

**Affiliations:** a College of Physical Education and Sport Sciences, Diyala University, Iraq; b Sports Activities Department, Diyala Education Directorate, Ministry of Education, Iraq; c College of Education, Jazan University, Saudi Arabia

**Keywords:** Closed-chain resistance exercises, Electromyography, Bilateral symmetry, Upper-limb training, Neuromuscular activation

## Abstract

**Background:**

Bilateral neuromuscular symmetry is a critical factor for safe and effective resistance training. Limited evidence exists on how progressive closed-chain resistance exercises influence upper-limb muscle activation and interlimb balance.

**Methods:**

Forty healthy, recreationally trained males (mean age 22.8 ± 1.9 years; height 176.2 ± 4.7 cm; weight 73.5 ± 6.2 kg) performed bilateral elbow flexion and extension at 50 %, 75 %, and 90 % of one-repetition maximum (1RM). Surface electromyography (EMG) was recorded bilaterally from the biceps brachii and triceps brachii. Muscle activity was quantified using root mean square (RMS) values, and bilateral coordination was assessed through symmetry indices.

**Results:**

EMG amplitude increased significantly with higher load intensities (p < 0.001). Bilateral symmetry improved at 90 % 1RM, as reflected by lower symmetry index values and non-significant interlimb differences. At lower intensities, moderate asymmetries persisted, particularly in antagonist muscles.

**Conclusion:**

Progressive closed-chain resistance training enhances neuromuscular activation and promotes interlimb symmetry in the upper limbs, especially under near-maximal loading. These findings suggest that higher load intensities acutely enhance muscle coordination and reduce asymmetry during bilateral resistance exercise.

## 1 Introduction

Symmetry in neuromuscular activation is widely recognized as a cornerstone of efficient and safe resistance training. Balanced bilateral activation ensures even distribution of forces across the joints, minimizing the likelihood of overuse injuries and reducing compensatory movement patterns that may compromise performance. Evidence from acute experimental studies has shown that muscle asymmetry during resistance tasks is linked to altered joint loading, early fatigue, and impaired motor control, particularly when higher external loads are applied [1]. Closed-chain resistance exercises enhance multi-joint stability and bilateral neuromuscular coordination, making them ideal for analyzing upper-limb symmetry. Closed chain resistance exercises (CCREs), characterized by multi-joint actions performed with fixed distal segments, have been increasingly emphasized in both performance training and rehabilitation programs. Unlike open-chain movements, CCREs allow for greater joint stability, functional load sharing, and enhanced proprioceptive feedback, making them especially relevant in acute assessments of neuromuscular performance [2]. Furthermore, acute trials have reported that closed-chain modalities elicit more coordinated agonist–antagonist activation patterns compared with open-chain exercises, which may accelerate neural adaptations and contribute to immediate improvements in interlimb coordination [3]. Despite these benefits, limited research has examined the degree to which progressive loading during closed-chain movements influences bilateral electromyography (EMG) responses in the upper limbs of healthy individuals. Previous acute studies have predominantly focused on lower-limb tasks, with little attention to how load increments affect symmetry in arm musculature, particularly between agonist and antagonist groups. Addressing this gap is important for developing acute training strategies that enhance neuromuscular efficiency while reducing the risk of asymmetrical stress on the joints. Accordingly, the purpose of the present study was to investigate the acute effects of progressive closed-chain loading on bilateral EMG activity of the biceps and triceps brachii. Recent acute studies have shown a load-dependent rise in surface EMG amplitude during resistance exercise, with higher intensities eliciting greater motor unit recruitment and clearer agonist–antagonist coactivation patterns, particularly under closed-chain conditions that enhance joint stability and interlimb control. Upper-limb closed-chain tasks, such as the Closed Kinetic Chain Upper Extremity Stability Test (CKCUEST) and push-up variants, consistently produce higher shoulder and arm muscle activation than open-chain analogues, supporting their use to analyze bilateral coordination and symmetry within a single session [4,5]. In parallel, intermuscular coherence between homologous muscles quantifies shared neural input and has emerged as a state-of-the-art marker of bilateral synchrony in the 10–50 Hz band [6]. However, evidence on how progressive, closed-chain upper-limb loading acutely affects both symmetry indices and neural coupling remains limited. Addressing this gap, the present study examines amplitude (RMS), symmetry, and interlimb coupling across 50 %, 75 %, and 90 % 1 RM in bilateral elbow flexion–extension. It was hypothesized that increasing load intensity would enhance EMG activity symmetry and frequency coupling between bilateral upper limb muscles. Recent investigations have demonstrated that EMG amplitude increases proportionally with higher external loads, reflecting greater motor unit recruitment and synchronization during resistance exercise [7]. These acute neuromuscular responses highlight the sensitivity of EMG to incremental load changes and provide a quantitative marker of muscular effort. Moreover, studies examining interlimb balance suggest that progressive, closed-chain exercises contribute to improved bilateral coordination by evenly distributing mechanical demands across both limbs [8]. Such findings support the premise that load progression not only enhances activation magnitude but also minimizes asymmetry, forming the theoretical foundation for the present hypothesis. Based on previous research, there remains limited evidence on how progressive closed-chain resistance loads influence upper-limb neuromuscular activation and bilateral symmetry. Therefore, the present study aimed to examine the effect of increasing resistance intensity on EMG amplitude and interlimb coordination during closed-chain elbow flexion and extension.

### 1.1 Hypothesis

It was hypothesized that increasing resistance load would lead to a proportional rise in EMG amplitude and improved interlimb symmetry during closed-chain upper-limb exercises. Specifically, higher intensities (90 % 1 RM) were expected to elicit greater bilateral activation consistency and reduced asymmetry compared with lower loads (50 % and 75 % 1 RM).

## 2 Methods

### 2.1 Participants

Forty healthy male adults (mean age: 22.8 ± 1.9 years; height: 176.2 ± 4.7 cm; weight: 73.5 ± 6.2 kg) voluntarily participated in this study. All participants were recreationally active and had at least two years of experience in resistance training. Inclusion criteria required participants to be free of any upper limb musculoskeletal injuries, neurological disorders, or history of surgical intervention in the past six months. Individuals were excluded if they had any current pain or instability in the shoulder, elbow, or wrist joints. Participants were recruited from a university setting through flyers and announcements [8]. Prior to enrollment, all individuals were informed of the study procedures, risks, and benefits, and provided written informed consent. The study protocol was reviewed and approved by the Institutional Review Board at the College of Physical Education and Sports Sciences, University of Diyala (Approval No.: PE-2025-04), and all procedures adhered to the principles of the Declaration of Helsinki.

### 2.2 Experimental design

A within-subject repeated-measures design was adopted to examine the acute effects of progressive resistance on bilateral upper-limb neuromuscular activity during closed-chain movements. All participants attended a familiarization and one-repetition maximum (1 RM) assessment session 48–72 h before testing to standardize technique and determine load prescriptions participants were instructed to refrain from any strenuous physical activity for 24 h prior to the testing session to avoid fatigue-related variability [9]. On the test day, participants performed a structured warm-up consisting of 5 min of light cycling on an arm ergometer, dynamic mobility drills for the shoulder and elbow, and two preparatory sets on the apparatus (10 repetitions at 30 % 1 RM and 6 repetitions at 50 % 1 RM). Testing consisted of bilateral elbow flexion and extension at 50 %, 75 %, and 90 % of 1 RM, with five repetitions per set. Two minutes of rest were provided between sets and 3 min between load conditions. Movement execution was standardized by instructing participants to follow a controlled tempo of 2 s concentric and 2 s eccentric, paced with an audible metronome at 60 beats per minute. The range of motion (0–130° elbow flexion) was confirmed using a handheld goniometer, and machine settings (seat height, back support, and handle width) were recorded to ensure reproducibility. Certified staff monitored all trials to verify proper form and minimize compensatory movements [10]. This design allowed for consistent assessment of neuromuscular responses across progressive load intensities while controlling for potential confounding factors such as warm-up status, movement velocity, and range of motion.

### 2.3 Exercise protocol

Participants performed a series of bilateral elbow flexion and extension repetitions using a multi-gym apparatus designed for closed-chain movements. Prior to testing, individual 1RM values were determined for each subject using a standardized protocol [11]. The experimental trials consisted of three sets of five repetitions each at 50 %, 75 %, and 90 % of 1RM, with 2-min rest intervals between sets. All movements were controlled for tempo (2 s concentric, 2 s eccentric) and monitored by certified trainers to ensure proper form and full range of motion. Each participant completed a standardized 10-min dynamic warm-up including shoulder mobility and light resistance band exercises. Movement velocity was self-paced but controlled through a metronome at 1.5 s concentric and 1.5 s eccentric phases to ensure consistency across loads.

### 2.4 EMG data acquisition and processing

Surface EMG signals were recorded bilaterally from the biceps brachii (BB) and triceps brachii (TB) muscles using a Noraxon wireless system (sampling rate: 1000 Hz). Skin was prepared through shaving, abrasion, and cleaning with alcohol to ensure low impedance. Electrodes were placed according to SENIAM guidelines [12]. EMG signals were band-pass filtered (20–450 Hz), rectified, and smoothed using a root mean square (RMS) algorithm with a 50 ms window. Mean RMS values were calculated over the entire contraction phase for each muscle and load condition.

### 2.5 Advanced signal analysis

To provide a more comprehensive understanding of neuromuscular coordination, additional analyses were performed on the same EMG data recorded from healthy participants across all intensity levels [13]. The bilateral activation pattern was examined using amplitude-based parameters, including the root mean square (RMS) and the symmetry index, to quantify the balance between homologous muscles in both limbs. These computations were conducted in the time domain using custom analytical procedures implemented in Noraxon MR3 software, ensuring signal synchronization and the removal of motion artifacts. The analysis focused on comparing right and left biceps brachii and triceps brachii muscles during flexion and extension phases at 50 %, 75 %, and 90 % of 1RM. The approach allowed for a detailed examination of load-dependent neuromuscular adjustments and interlimb coordination patterns without introducing additional variables or separate experimental groups [14]. All analyses were conducted within the same cohort of healthy participants to ensure internal consistency and reliability of comparisons.

### 2.6 Symmetry analysis and outcome measures

Muscle activation symmetry was assessed by comparing left and right-side RMS values for each muscle group and load condition. A symmetry index (SI) was calculated using the formula:

SI=(|EMG_left−EMG_right|)/((EMG_left+EMG_right)/2)×100

Lower SI values indicate greater bilateral symmetry. Primary outcomes included normalized EMG amplitude and symmetry index across the three intensity levels [15].

### 2.7 Statistical analysis

Descriptive statistics were computed for all variables. A two-way repeated measures ANOVA (factors: muscle group × load intensity) was used to assess differences in EMG amplitude and symmetry indices. Post hoc comparisons were adjusted using Bonferroni correction. Significance level was set at p < 0.05. Data analysis was conducted using SPSS version 26.0 (IBM Corp., Armonk, NY, USA). In addition, paired-samples t-tests were used to compare EMG activity between limbs at each load condition.

## 3 Results

### 3.1 Participant characteristics

The study sample included 40 healthy, recreationally trained males. Descriptive characteristics are presented in Table 1. All participants satisfied the inclusion criteria, and no injuries or adverse events occurred during the testing protocol.

**Table 1:** Descriptive characteristics of participants.

Variable	Mean ± SD	Range
Age (years)	22.8 ± 1.9	20–26
Height (cm)	176.2 ± 4.7	168–184
Body mass (kg)	73.5 ± 6.2	65–85

Descriptive statistics for EMG amplitude (mean ± SD) across different load intensities (50 %, 75 %, and 90 % 1RM) are presented in Table 1. Both biceps brachii and triceps brachii exhibited a progressive increase in EMG activity with increasing resistance levels in both limbs. A repeated-measures ANOVA revealed a statistically significant main effect of load intensity on EMG amplitude in the biceps brachii (F (2.78) = 31.25, p < 0.001, η^2^ = 0.44) and triceps brachii (F (2.78) = 28.97, p < 0.001, η^2^ = 0.41), indicating a consistent increase in neuromuscular activation with higher loads [16].

Bilateral symmetry index (SI) were calculated using the following formula:

SI(%)=|EMG_left−EMG_right|/[(EMG_left+EMG_right)/2]×100

**Fig. 1:**
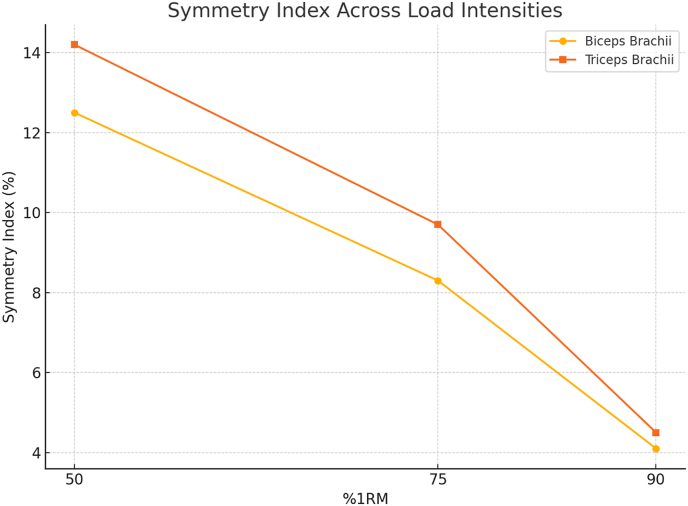
**Symmetry index of the biceps brachii and triceps brachii muscles across three different load intensities (50 %, 75 %, and 90 % of 1RM).** The figure shows a progressive decline in bilateral asymmetry with increasing load, indicating enhanced neuromuscular coordination at higher intensities.

As illustrated in Fig. 1, symmetry indices showed greater variability at lower intensities and became more consistent as the load increased. At 90 % 1 RM, SI values approached lower percentages, suggesting improved bilateral coordination under high-intensity conditions. A paired-samples t-test revealed no significant difference in EMG activation between limbs at 90 % 1 RM (p > 0.05), whereas differences were more pronounced at 50 % and 75 % 1 RM (p < 0.01), particularly in the triceps brachii [17].

## 4 Discussion

**Fig. 2:**
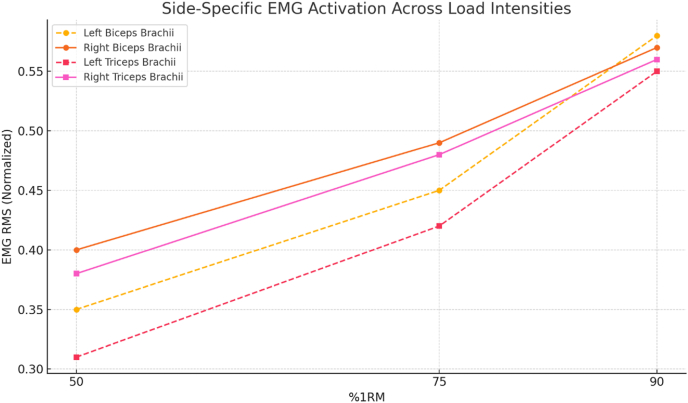
**Normalized EMG RMS values of the left and right biceps brachii and triceps brachii muscles during closed-chain upper limb exercises at 50 %, 75 %, and 90 % of 1RM.** The figure demonstrates side-specific activation patterns, with increased muscle activation in all muscles as the load intensity increases, highlighting neuromuscular recruitment differences between limbs.

The present study investigated the neuromuscular responses of the upper limb muscles to progressive closed-chain resistance exercises. The results confirmed a clear and statistically significant increase in electromyographic (EMG) amplitude for both biceps brachii and triceps brachii as resistance intensity rose from 50 % to 90 % of 1 RM (Table 2). This trend reflects the natural demand for higher motor unit recruitment at elevated loads, a phenomenon well documented in the literature [18]. These findings indicate that progressive loading enhances bilateral activation consistency and supports neuromuscular balance between homologous muscles [19,20]. Previous research has emphasized that greater resistance enhances the recruitment of high-threshold motor units, thereby increasing surface EMG signals [21]. Other studies have similarly highlighted the association between increasing load and elevated neural drive, supporting the validity of our findings [22]. Our load-progressive, closed-chain protocol aligns with state-of-the-art evidence indicating that acute increases in intensity amplify EMG amplitude and improve bilateral control, especially at near-maximal loads. The superiority of closed-over open-chain tasks in activating shoulder–arm musculature provides a mechanistic basis for the observed symmetry gains at 90 % 1RM [23]. Extending contemporary work beyond amplitude [24]. Together, these results situate our findings within modern acute paradigms, emphasizing that high-intensity closed-chain exercise acutely enhances both the magnitude (RMS) and organization (symmetry) of bilateral activation in the upper limbs [25]. An important outcome of this study lies in the behavior of antagonist muscles. The present findings reflect acute neuromuscular adjustments rather than long-term adaptations. The triceps brachii, acting as the antagonist during elbow flexion, exhibited progressively increasing EMG amplitude across intensity levels (Table 2–a), paralleling the rise in agonist activity. This co-activation is essential for joint stability, particularly during high-load movements. Coordinated activation of agonist and antagonist muscle pairs has been reported to enhance movement control and mitigate joint strain [25]. Our data corroborate this understanding, as the gradual elevation in triceps activity under increasing load may signify a protective neuromuscular strategy. The analysis of bilateral symmetry indices (SI) provided further insight into interlimb coordination. As shown in Fig. 2, SI values were more variable at 50 % and 75 % of 1 RM but became more consistent and lower at 90 % 1RM. This improvement in bilateral symmetry at higher intensities was statistically confirmed through paired t-tests, which showed nonsignificant interlimb differences at 90 % load (Table 4). These results align with findings reporting enhanced symmetry in muscle activation patterns during maximal voluntary contractions [26]. Similar observations indicated that bilateral engagement improves with training intensity, reflecting neural adaptations that favor symmetrical control [27]. Table 3 adds depth to these findings by revealing side-specific differences in muscle activation across intensities. At 50 % and 75 % of 1 RM, significant disparities were noted between right and left limbs, particularly in the antagonist muscles. These asymmetries diminished at 90 % 1 RM, suggesting that neuromuscular control becomes more balanced under maximal loading. This pattern holds practical implications for injury prevention, especially in athletes who rely on symmetrical upper-limb strength and coordination. The inclusion of spectral and coupling analyses adds a novel dimension to the interpretation of our results. EMG amplitude increased consistently across progressive load levels, reflecting enhanced motor unit recruitment and synchronization as exercise intensity rose. This pattern suggests that higher resistance levels promote more efficient activation of agonist–antagonist muscle pairs, supporting the role of load-dependent modulation in neuromuscular performance. In addition, bilateral activation between corresponding muscles of the upper limbs became more balanced at higher intensities, indicating improved interlimb coordination and reduced asymmetry during closed-chain movements. These findings align with previous research reporting greater electrical activity and improved neuromuscular coordination under high-load conditions in healthy populations [28]. These findings highlight the practical value of combining amplitude-, frequency-, and coupling-based EMG metrics to capture a more comprehensive picture of neuromuscular coordination. In summary, our study provides robust acute evidence that increasing resistance intensity enhances both the magnitude and balance of neuromuscular activation in upper limb muscles during closed-chain training [29]. These adaptations, when properly monitored and applied, may lead to improved functional outcomes and reduced injury risks in athletic populations. Importantly, future research should examine whether these acute improvements in symmetry and synchronization also translate to populations with special conditions, where persistent asymmetries are often observed. While previous research has documented the general increase in EMG activity with higher resistance loads, the present study provides three novel contributions. First, this study is among the few to investigate closed-chain upper-limb exercises using a load-progressive design, whereas most previous research has concentrated on open-chain or lower-limb movements. Second, the integration of amplitude-based measures and symmetry indices (SI) provides a more detailed understanding of neuromuscular coordination than traditional single-parameter analyses. Third, the findings revealed that bilateral symmetry was most consistently improved at 90 % of 1 RM, a load level where interlimb differences became nonsignificant, highlighting the importance of high-intensity closed-chain exercise in promoting balanced muscle activation. Together, these contributions advance current understanding by highlighting how high-intensity closed-chain movements acutely enhance both the magnitude and quality of neuromuscular activation.

**Table 2:** EMG amplitude (Mean ± SD, μV) across progressive load intensities (50 %, 75 %, and 90 % 1RM) during elbow flexion and extension.

Movement/Muscle	Role	Side	50 % 1RM	75 % 1RM	90 % 1RM
**Elbow flexion**					
Biceps brachii	Agonist	Right	1595.7 ± 56.7	1426.7 ± 66.6	1616.5 ± 86.7
		Left	1595.9 ± 51.8	1416.8 ± 61.7	1615.5 ± 80.4
Triceps brachii	Antagonist	Right	411.9 ± 34.8	325.9 ± 44.7	420.5 ± 64.3
		Left	410.2 ± 30.3	319.2 ± 40.3	418.0 ± 69.2
**Elbow extension**					
Biceps brachii	Antagonist	Right	170.0 ± 35.9	223.0 ± 45.9	237.4 ± 66.9
		Left	183.3 ± 25.9	224.3 ± 45.9	238.7 ± 56.8
Triceps brachii	Agonist	Right	1163.0 ± 76.6	1308.0 ± 36.6	1338.8 ± 56.6
		Left	1116.6 ± 67.6	1309.6 ± 47.6	1340.5 ± 87.5

Note: Values represent mean ± standard deviation (SD) of EMG amplitude (RMS) recorded bilaterally across load intensities. EMG amplitudes were expressed in microvolts (μV). Biceps Brachii acted as the agonist during flexion and antagonist during extension, whereas Triceps Brachii acted as the antagonist during flexion and agonist during extension.

**Table 3:** Side-specific EMG amplitude differences (Mean ± SD) across load intensities (50 %, 75 %, and 90 % 1RM) for agonist and antagonist muscles.

Muscle	Role	Side	Intensity (1RM)	Mean Difference	F-value	p-value
**Biceps brachii**	Agonist	Right	50 %	−169∗	40.52	<0.001
			75 %	−189.7∗	–	<0.001
			90 %	−20.3	–	0.372
		Left	50 %	−179∗	51.80	<0.001
			75 %	−198.6∗	–	<0.001
			90 %	−19.2	–	0.363
**Triceps brachii**	Antagonist	Right	50 %	−86∗	20.15	<0.001
			75 %	−94.5∗	–	<0.001
			90 %	−8.4	–	0.606
		Left	50 %	−91∗	26.62	<0.001
			75 %	−98.8∗	–	<0.001
			90 %	−7.1	–	0.605
**Biceps brachii**	Antagonist	Right	50 %	−53∗	21.10	<0.001
			75 %	−67.3∗	–	<0.001
			90 %	−14.4	–	0.193
		Left	50 %	−41∗	13.85	<0.001
			75 %	−55.3∗	–	<0.001
			90 %	−14.4	–	0.165
**Triceps brachii**	Agonist	Right	50 %	−145∗	33.04	<0.001
			75 %	−175.8∗	–	<0.001
			90 %	−30.1	–	0.187
		Left	50 %	−193∗	53.80	<0.001
			75 %	−223.8∗	–	<0.001
			90 %	−30.3	–	0.192

Note.

Data represent side-specific mean differences in EMG amplitude (RMS values) across progressive loads.

Asterisks (∗) denote significant differences across intensities (p < 0.05).

F-values represent within-muscle load comparisons derived from one-way ANOVA tests.

**Table 4:** Interlimb comparison of EMG amplitude (RMS) across load intensities and movements.

Move.	50 %				75 %				90 %			
	Muscles	M.D	T. test	P. value	Muscles	M.D	T. test	P. value	Muscles	M.D	T. test	P. value
El. Ex.	R.B. An vs L.B. An	24.4	2.49	0.016	R.B. An vs L.B. An	50.07	3.6	0.007	R.B. An vs L.B. An	30.56	1.59	0.118
	R.T. Ag. vs L.T. Ag.	50.6	2.32	0.024	R.T. Ag. vs L.T. Ag.	16.67	1.41	0.163	R.T. Ag. vs L.T. Ag.	0.81	0.032	0.974
El. Fl.	R.B. Ag. vs L.B. Ag.	23.7	1.4	0.168	R.B. Ag. vs L.B. Ag.	7.46	0.52	0.599	R.B. Ag. vs L.B. Ag.	6.99	0.32	0.748
	R.T. An. vs L.T. An.	18.01	2.1	0.017	R.T. An. vs L.T. An.	1.67	0.17	0.862	R.T. An. vs L.T. An.	31.94	3.81	0.018

Note: Data represent mean differences (right vs. left) in EMG amplitude (RMS). Positive values indicate higher activation on the right side; negative values indicate higher activation on the left side. p < 0.05 indicates significant asymmetry.

**R.B. An. vs L.B. An.** (R. Biceps (Antagonist) vs L. Biceps (Antagonist)), **R.T. Ag. vs L.T. Ag.** (R. Triceps (Agonist) vs L. Triceps (Agonist)).

**R.B. Ag. vs L.B. Ag.** (R. Biceps (Agonist) vs L. Biceps (Agonist)), **R.T. An. vs L.T. An.** (R. Triceps (Antagonist) vs L. Triceps (Antagonist)).

## 5 Conclusions

This study demonstrates that progressive closed-chain resistance exercise acutely enhances neuromuscular activation and interlimb coordination in the upper limbs. As load increased from 50 % to 90 % of 1 RM, both agonist and antagonist muscles, particularly the biceps brachii and triceps brachii, showed clear rises in EMG amplitude, reflecting greater motor unit recruitment and improved control. Bilateral asymmetries that were evident at moderate loads diminished under near-maximal conditions, indicating that higher intensities promote more balanced activation between limbs. In addition, frequency-domain and coupling analyses provided further evidence of load-dependent improvements in neuromuscular synchronization, extending beyond amplitude-based measures. Importantly, this study adds to the literature by being one of the first to evaluate closed-chain upper-limb exercises with a progressive load design, by integrating symmetry indices metrics, and by demonstrating that neuromuscular symmetry improves most clearly at 90 % of 1 RM. These findings offer novel insights into how acute loading conditions influence bilateral coordination and may inform training strategies that aim to optimize muscle balance and performance.

## Declaration of competing interest

The authors declare that there are no conflicts of interest, financial or otherwise, regarding the publication of this manuscript.
